# Effects of proprioceptive training on sports performance: a systematic review

**DOI:** 10.1186/s13102-024-00936-z

**Published:** 2024-07-04

**Authors:** Osman Yılmaz, Yusuf Soylu, Nurtekin Erkmen, Turgut Kaplan, Ladislav Batalik

**Affiliations:** 1 School of Physical Education and Sports, Osmaniye Korkut Ata University, Osmaniye, Turkey; 2 Faculty of Sports Sciences, Tokat Gaziosmanpasa University, Tokat, Turkey; 3 Faculty of Sports Sciences, Selcuk University, Konya, Turkey; 4 None Department, Aksaray, Turkey; 5 Department of Physiotherapy and Rehabilitation, Faculty of Medicine, Masaryk University, Brno, Czech Republic; 6 Department of Rehabilitation, University Hospital Brno, Brno, Czech Republic; 7 Department of Public Health, Faculty of Medicine, Masaryk University, Brno, Czech Republic

**Keywords:** Athletic performance, Proprioceptive exercise, Sports rehabilitation, Sports performance, Sports and proprioception

## Abstract

**Background:**

Proprioception, the ability to sense the body’s position and movement, is essential for athletic performance and physical well-being. The literature highlights the importance of proprioceptive training in rehabilitation, sports performance, injury prevention, and motor function enhancement. Targeted training programs can improve balance, coordination, motor learning, and overall physical performance. This systematic review aimed to examine the effects of proprioceptive training methods on sports and athletic performance.

**Methods:**

A comprehensive search was conducted using the Web of Science, PubMed, and Scopus databases, and a literature review was performed based on the PICO criteria outlined in the abstract and title.

**Results:**

Following the search, 178 articles were identified using relevant keywords, of which 19 directly addressed sports performance and were included in this study. The findings revealed that proprioceptive training had a positive influence on various aspects of athletic performance, including physiological capacity, balance, explosive strength, speed, agility, postural stability, knee joint position sense, muscle activation, reduction of chronic joint instability, dribbling, passing, and technical ball-control skills.

**Conclusions:**

These results indicate that proprioceptive training can be an effective strategy for experts and coaches to enhance athletes’ physical performance. Primarily, proprioceptive exercises should be used inside and outside the training sessions to enable athletes to interact more effectively with their bodies, reduce the risk of injury, and improve power transfer.

**Supplementary Information:**

The online version contains supplementary material available at 10.1186/s13102-024-00936-z.

## Introduction

Body movements are regulated by the somatosensory and sensorimotor systems, which work together to provide efficient proprioception and a sense of joint position awareness that goes beyond the feeling of movement [1]. Proprioception is the capacity to sense and comprehend the body’s position and movement, encompassing balance, motion, and environmental navigation [2, 3]. Therefore, improving movement patterns is vital for improving body awareness and coordination, which can significantly enhance motor skills [4, 5]. Several studies have reported the effects of proprioceptive training on balance, trunk control, gait speed, essential functional mobility [6], motor, somatosensory, and sensorimotor functions [4], balance [7], and knee joint function [8]. Proprioceptive training and exercise specialisation can improve physical performance and reduce injury prevention in sports.

Proprioception significantly affects an individual’s ability to excel in sports, a crucial aspect of athletic performance [7, 9, 10]. Proprioceptive training is a form of exercise that focuses on improving proprioception, which is the body’s ability to sense and interpret stimuli regarding position, movement, and balance [11]. Therefore, understanding body positioning and movements to improve performance, injury prevention, and overall playing skills has become increasingly important. Proprioceptive training is an effective method for running, changing direction, jumping, turning on one leg, stabilizing joints, activating joint position and muscle control, preventing and rehabilitating injuries, and improving motor performance [4, 5]. In conclusion, as proprioceptive training helps athletes adapt to changing situations [12], coaches and performance practitioners focus on improving balance, stability, and body control, which are crucial for athletes to perform optimally in the field.

Proprioception in sports is widely recognised for its role in injury prevention, rehabilitation, talent identification, and performance enhancement [13]. Improved balance, which is closely linked to proprioception, is associated with enhanced athletic performance and reduced lower limb injuries [14]. Research indicates that Proprioceptive training can stabilise joints, prevent injuries, and improve dynamic neuromuscular control in athletes, thereby positively affecting their performance [15, 16]. Proprioceptive training enhances dynamic neuromuscular control in athletes such as fencers by stabilising the ankle joint and preventing injuries [7]. Moreover, core proprioceptive training has been found to improve the quality of executive functions in young female basketball players, highlighting the broader cognitive benefits of proprioceptive training in sports performance [17]. Furthermore, proprioceptive training has been associated with improved shoulder stability, throwing performance in water polo, and shoulder proprioceptive acuity, which is crucial for achieving higher sports performance levels [18, 19]. Proprioception accuracy is crucial for knee stability, efficient movement execution, and injury prevention in various sports [20, 21]. Moreover, long-term neuromuscular training positively impacts ankle joint position sense in athletes, highlighting the enduring benefits of proprioceptive training in sports [22].

The field of sports science necessitates further exploration and study concerning proprioceptive training due to the current lack of comprehensive research in this area. Despite the recognized significance of proprioception in sports performance and injury prevention, more targeted research is needed to address specific knowledge gaps and enhance practical applications. Lauersen et al. [23] conducted a systematic review and meta-analysis of exercise interventions for preventing sports injuries, revealing that proprioceptive training reduces injury risks more effectively than interventions such as stretching, underscoring its potential importance in sports injury prevention strategies. Federici et al. [24] identified common flaws in proprioceptive training in sports, paving the way for future improvements in training methodologies. McBain et al. [25] highlighted the lack of research on intermediate outcomes in non-contact sports and upper extremity injuries, emphasizing a critical gap in current knowledge. This underscores the need for more studies on applying proprioceptive training in various sports contexts to address the injury risks associated with upper-extremity movements. The primary objective of this study was to systematically document various interventions employed to improve sports performance. Additionally, we aimed to emphasize the methods used to quantify the impact of proprioceptive training on sports performance and evaluate the efficacy of proprioceptive training as a tool in athletic settings for improving motor function and performance.

## Materials and methods

### Objective

This study presents a systematic review of peer-reviewed scientific articles investigating the effects of proprioceptive training on athletic performance characteristics in athletes.

### Protocol and registration

**Fig. 1:**
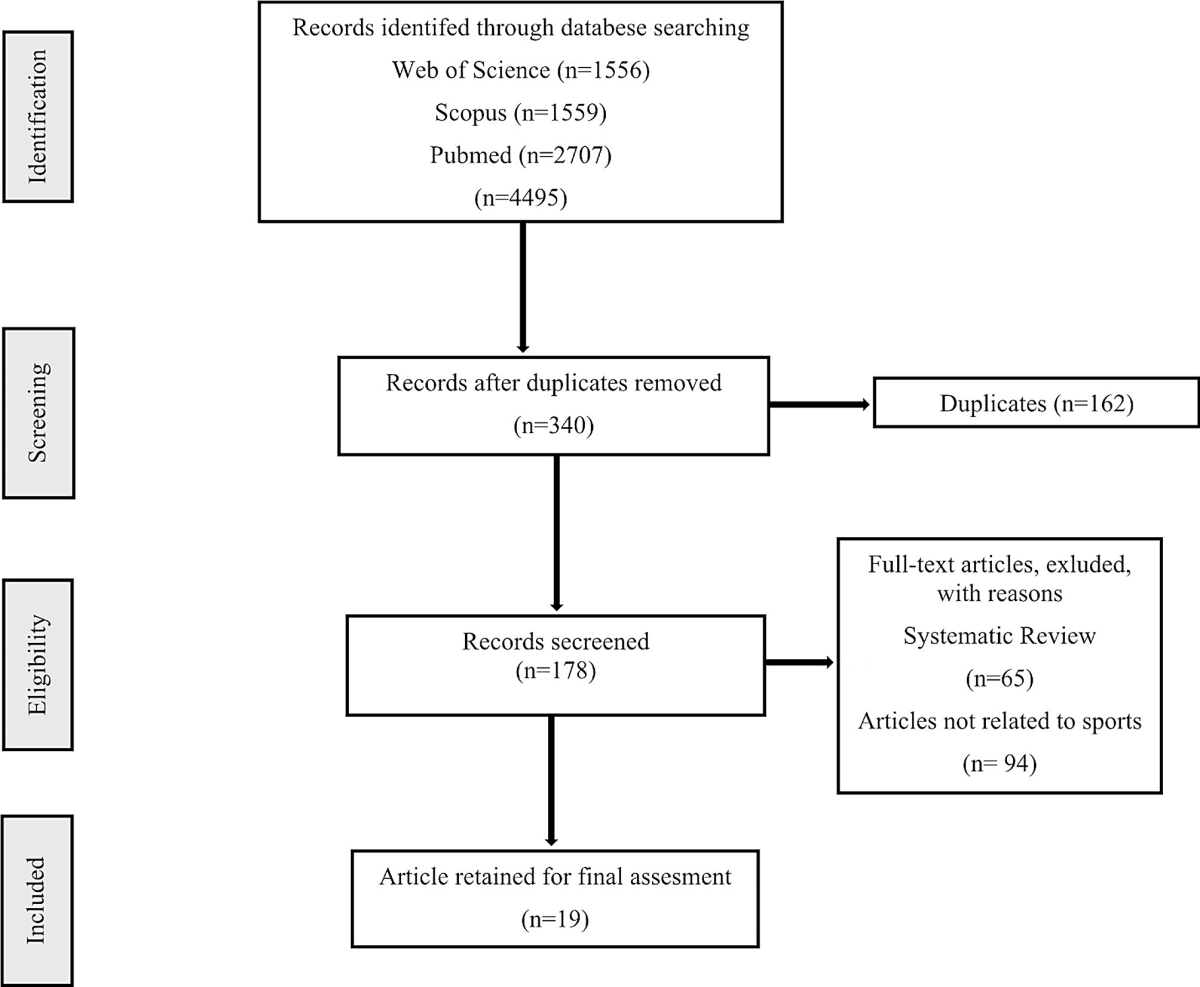
PRISMA flow diagram template for systematic reviews. The new design is adapted from flow diagrams proposed by Moher

The systematic literature review followed the Preferred Reporting Items for Systematic Reviews and Meta-Analyses (PRISMA) guidelines (Fig. 1) [26].

### Eligibility criteria

This study presents a systematic review of peer-reviewed scientific articles investigating the effects of proprioceptive training on athletic performance characteristics in athletes. This review focused solely on studies examining the impact of proprioceptive training interventions or training on athletes’ sports performance, encompassing functional and structural aspects within their research protocols. Only experimental studies were included in this study. No limits were placed on the sex, age, sports type, or athletic level of eligible articles. This study only included athletic participants. We had articles that utilised applied methodologies to provide perspective.

### Search strategy

A comprehensive search was conducted using a combination of specific keywords likely to be found in the titles or abstracts of studies investigating the sports performance of proprioceptive training. The research comprehensively searched relevant scientific studies published between 01 January 2010 and 01 October 2022 in the Web of Science (WOS), PubMed, and Scopus databases. Keywords were grouped into two distinct categories: those designed to retrieve studies utilising proprioceptive training intervention as an exercise technique or objective (encompassing terms like “central stabilization”, “eyes group exercise”, and “feet group exercise”) and those focused on the balance or injury risk of proprioceptive training intervention. Second, the physiological, physical, and technical outcomes of proprioceptive training include dribbling, explosive power, and strength. This study used ‘’*proprioceptive training*’’, ‘’*proprioceptive exercise* and ‘’*proprioception training’’* The reference lists of the identified articles were thoroughly examined.

### Eligibility assessment

Two assessors were assigned to each intervention (see the authors’ contributions). Two assessors evaluated the articles separately by examining titles and abstracts to determine if they met the criteria for inclusion in the analysis. The criteria were based on four factors: population, intervention, comparison, and outcome (PICO). The assessors participated in a discussion and agreed to any disagreements regarding including publications. Subsequently, the two authors evaluated the complete copies of the listed articles based on the same criteria to determine their final eligibility (See Table 1).

**Table 1:** TESTEX methodological quality assessment

Studies	Criteria												Total TESTEX Point	Methodological quality
	1	2	3	4	5	6	7	8	9	10	11	12		
Gidu et al., 2022	1	0	0	1	0	1	0	2	1	1	0	1	9	Excellent
Souglis et al., 2022	1	0	0	1	0	1	0	2	1	1	0	0	8	Excellent
Harry-Leite et al., 2022	1	1	0	0	0	1	0	1	1	0	0	1	7	Good
Viran&Canlı, 2022	1	0	0	0	0	1	0	2	1	1	1	1	9	Good
Achilleopoulos et al., 2022	1	0	0	0	0	1	0	2	1	1	0	0	6	Moderate
Beydağı & Talu, 2021	1	0	0	0	0	1	0	1	1	0	1	1	7	Good
Antohe et al., 2020	1	0	0	1	0	1	0	1	1	0	0	0	6	Moderate
Domeika et al., 2020	1	0	0	0	0	1	0	1	1	1	0	1	7	Good
Rhodes et al., 2020	1	1	0	0	0	1	0	2	1	1	0	0	7	Good
Zacharakis et al., 2020	1	1	0	1	0	1	0	2	1	1	0	0	8	Good
Yoo et al., 2018	1	1	0	1	0	2	0	2	1	1	0	1	11	Good
De Vasconcelos et al., 2020	1	0	0	0	0	1	0	2	1	1	0	1	8	Good
Ondra et al., 2017	1	0	0	1	0	2	0	2	1	1	0	1	10	Good
Moreria et al., 2017	1	1	0	1	0	1	0	2	1	0	0	0	8	Moderate
Winter et al., 2014	1	0	0	1	0	1	0	2	1	1	0	0	7	Moderate
Zouita et al., 2013	1	0	0	1	0	1	0	1	1	1	0	0	6	Good
Romero-Franco et al., 2013	1	1	0	1	0	1	1	2	1	1	1	1	11	Good
Nikolaos et al., 2012	1	0	0	1	0	1	0	2	1	1	0	0	7	Moderate
Evangelos et al., 2012	1	0	0	1	0	1	0	2	1	1	0	0	7	Moderate

TESTEX scale criteria: 1; Eligibility criteria specified (1 point), 2; Randomization specified (1 point), 3; Allocation concealment (1 point), 4; Groups similar at baseline (1 point), 5; Blinding of assessor (1 point), 6; No point – if withdrawals are >15%, 1 Point – if adherence>85%, 1 Point – if adverse events are reported, 1 Point – if exercise attendance is reported, Total possible (3 point), 7; Intention-to-treat analysis (1 point), 8; 1 Point – if between-group statistical comparisons are reported for the primary outcome measure of interest, 1 Point – if between-group statistical comparisons are reported for at least one secondary outcome measure, Between-group statistical comparisons reported (2 point, 9; Point measures and measures of variability for all reported outcome measures (1 point), 10; Activity monitoring in control groups (1 point), 11; Relative exercise intensity remained constant (1 point), 12; Exercise volume and energy expenditure (1 point). Quality points; < 4 as “poor”, 4–7 as “moderate”, 8–11 as “good” and > 11 as excellent

### Outcome measures

The primary outcome of interest was to evaluate changes in the measured performance parameters of proprioceptive training practices, as measured by official or validated instruments, before and after the intervention. This review included 19 studies investigating the effects of proprioceptive training on sports performance as an independent variable of the intervention strategy (Table 3). Five hundred fifty-six participants from different branches, aged between 12 and 24 years, participated in this study. Most studies have focused on the effects of proprioceptive training on balance, postural stability, knee joint position sense, muscle activation, and the reduction of chronic joint instability. Some studies have investigated its effects on flexibility, explosive strength, speed, agility, dribbling, passing, and technical ball control skills.

### Data extraction

Following the data search, the relevant data were obtained based on the study format. This comprised the following information: (1) author’s name, nationality, and year of publication; (2) sample group; (3) treatment group; (4) duration of intervention; (5) certified instructor; (6) outcome measures; and (7) results. The data were then structured into a standard format, and a database was developed by one reviewer, who subsequently verified it with the help of two additional reviewers.

### Quality assessment

The methodological quality of the RCTs was assessed using the Testex Scale [27]. The Testex scale, which consists of 12 items, was developed to evaluate the methodological quality of randomized controlled trials (RCTs). Scores < four were considered “poor quality”, scores between 4 and 7 were considered “moderate quality”, scores between 8 and 11 were considered “good quality”, and scores above 11 were considered “excellent quality” [28]. (Table 2).

**Table 2:** PICO criteria

PICO	Criteria
Population	Athletes
Intervention	Proprioceptive training application for athletes
Comparison	Control group The experimental group (proprioceptive training, central stabilization, regular training, eyes group exercise, feet group exercise)
Outcome	Physical and physiological responses (development of technical skill (dribbling, passing juggling ball), speed, explosive strength, maximal strength, agility, balance, postural stability, shoulder stability, knee joint position sense, muscle activation, reduction of chronic joint instability and physiological capacity)

## Results

### Study selection

The initial search yielded many articles, necessitating rigorous exclusion criteria applied consistently by two independent reviewers. Despite these efforts, the field’s diverse terminology and keyword usage have resulted in many irrelevant studies (see Fig. 1). This finding highlights the challenges associated with conducting systematic reviews of this field. Data were synthesised and standardised after retrieving relevant studies through a database search. A standardised search yielded studies from two databases with a significant overlap that fulfilled the inclusion criteria. Careful comparison of the merged references: Only published studies were included. This study was limited to peer-reviewed journals that were published in English. A systematic review of the literature was performed following the Preferred Reporting Items for Systematic Reviews and Meta-Analyses (PRISMA) guidelines [26] (Fig. 1). After obtaining the article results from the databases, titles and abstracts were examined to identify articles related to the keywords. The criteria for including articles in this review were determined using the PICO (Population, Intervention, Comparison, Outcome) approach [29] (Table 2). Following the search, 178 articles were identified using acceptable keywords. Of these, 19 research articles specifically focused on sports performance were selected for inclusion in this study (Fig. 1).

### Study characteristics

Overall, the study included 621 participants between the ages of 12 and 24 who engaged in various sports, including soccer (263), basketball (132), handball (22), volleyball (18), taekwondo (30), skaters (28), fencing (19) and athletes (109). This study aimed to examine the acute effects of proprioceptive training. The application times for the remaining 18  studies varied from a minimum of six weeks to a maximum of 20 weeks. These studies show that weekly training days range from 3 to 5 days. The duration of the proprioceptive training sessions ranged from 10 to 60 min. One study was conducted as an acute effect. The measurement methods used in these studies and the results obtained are explained in detail in Table 3.

**Table 3:** Physical and physiological responses to proprioceptive training practice

Reference	Sample	Treatment Group	Duration of Intervention (weeks/frequency)		Certificated Instructor	Outcome Measure	Results
Gidu et al., 2022	Male Soccer Players (*N*=96)	EG=48 (14.2±0.4) CG=48 (14.0±0.0)	8 weeks	2 days/30 min	NA	Static Balance Test Vertical, Horizontal, Lateral Jumping Test Arrowhead Agility Test Short Dribbling Test	Proprioceptive training significantly improved balance, explosive strength, agility, and dribbling skills in male football players.
Souglis et al., 2022	Female Soccer Players (*N*=48)	IG=24 (23.88±3.01) CG=24 (24.4±2.84)	16 weeks	5 days/20 min	NA	Body Fat Measures VO2 Max Test Agility Ladder Drills Test Illinois Agility Test Juggling, Heading, Shooting, Passing, Dribbling, Dribbling and Passing Tests	Proprioceptive training has improved the physiological capacities and shooting, short and long passing, heading front and side, and juggling technical skills of female soccer players.
Harry-Leite et al., 2022	Male Athletes (*N*=60)	SP=30 (19.4±1.2) BP=30 (20.1±2.4)	Acute Effects	NA	YES	Balance Error System Score Test Y-Balance Test Assessment of Joint Position Sense	The significance of both proprioceptive and non-specific exercise sessions in enhancing knee joint position sense and balance cannot be overstated. However, proprioceptive exercises are more effective at improving joint position sense than non-specific exercises.
Viran & Canlı, 2022	Soccer Players (*N*=30)	PTG=11 (NA) CG=10 (NA)	8 weeks	3 days/25–30 min	NA	20-Meter Sprint Test Pro-Agility Test Countermovement Jump Test Core Endurance Test Alternate Wall Toss Test Y Balance Test Mor-Christian General Soccer Ability Skill Test Loughborough Test	There was no significant improvement in hand-eye coordination, vertical jump, core endurance, agility, sprint and balance performance. A significant improvement was observed in pass (shot) and ball control technical performance characteristics.
Achilleopoulos et al., 2022	Youth Female Volleyball Players (*N*=18)	EG= 10 (NA) CG=8 (NA)	8 weeks	3 days/ 19–28 min	NA	Dynamic Balance Test Technical Skill Tests	8-week proprioception training improved dynamic balance and service and passing technical skills in volleyball players.
Beydağı & Talu, 2021	Professional Male Soccer Players (*N*=20)	PG=20 (20.55±3.55)	6 weeks	3 days/ 10–15 min	YES	Static and Dynamic Balance Tests	Proprioceptive exercises provided improvement in all of the static balance parameters of the football players. In the dynamic balance parameters, it was seen that there was an improvement in other parameters except the one-leg stance position.
Antohe et al., 2020	Junior Handball Players (*N*=22)	EG= 22 (NA)	15 weeks	3 days/15–20 min	NA	Muscle Coactivation Index Chronic Joint İnstability Assessment	Proprioceptive exercises were found to be effective in reducing chronic joint instability and increasing muscle coactivation values in handball players.
Domeika et al., 2020	Basketball Players (*N*=31)	BTG=17 (NA) CG=14 (NA)	8 weeks	3 days/ 20 min	NA	Y Balance Test Postural Stability Test	Proprioceptive training program has improved the balance of basketball players.
Rhodes et al., 2020	Soccer Players (*N*=16)	PTG=8 (NA) CT=8 (NA)	16 weeks	5 days/10 min	NA	Biodex Dynamic Stability Test	Proprioceptive training program has improved the dynamic balance of football players.
Zacharakis et al., 2020	Basketball Players (*N*=55)	EB Boys=15 (13.2±0.2) CG Boys=15 (13.2±0.1) EG Girls=13 (13.2±0.2) CG Girls=12 (13.2±0.2)	8 weeks	3 days/14–26 min	NA	Dynamic and Static Balance Test Speed and Accurate Shooting Test Passing Accuracy Test Obstacle Dribbling Test Defensive Sliding Test Lay-Up Test No Ball Maneuver Running Test	It was observed that 8 weeks of proprioceptive training improved passing accuracy in boys and girls, static balance and fast shooting in boys, and dynamic balance in girls.
De Vasconcelos et al., 2020	Fencing Athletes (*N*=19)	IG=10 (16.80±2.34) CG=9 (24.00±6.65)	12 weeks	3 days/30 min	NA	Star Excursion Balance Test	12-week Proprioceptive training program was able to improve dynamic neuromuscular control in fencing athletes.
Yoo et al., 2018	Taekwondo Athletes (*N*=30)	PGT=10 (20.0±2.6) MSTG=10 (19.2±0.8) CG=10 (19.1±0.7) All Groups= 8 male, 2 females consisted	6 weeks	3 days/ 60 min	NA	Balance Test	Proprioceptive and muscle strength training have been shown to improve athletic performance and improve athletes’ skill levels in maintaining the taekwondo crane stance.
Ondra et al., 2017	Male Youth Basketball Players (*N*=20)	IG=10 (17.3±1.3) CG=10 (16.5±1.8)	20 weeks	3 days/20 min	NA	Lower Limb Dominancy Test Balance Stability Test	Proprioceptive and neuromuscular training specifically for basketball players improved postural stability in both the dominant and non-dominant limbs.
Moreria et al., 2017	Young Soccer Players (*N*=24)	PTG=12 (15.60±0.50) CST=12 (15.32±0.51)	9 weeks	3 days/16 min	NA	Square Agility Test Sit Up Abdominal Strength Test Side Hope Balance Test Well Banks Flexibility Test Shuttle Run Speed Test	Speed performance improved in both groups, but agility performance only improved in the proprioceptive training group. There was no significant difference in balance, abdominal strength, and flexibility performance between the two groups.
Winter et al., 2014	Young Speed Skaters (*N*=28)	IG=14 (12.6±1.5) CG=14 (12.9±1.7)	12 weeks	5 days/15 min	NA	Dynamic balance test (Biodex System)	12-week Proprioceptive training program improved dynamic balance in young speed skaters
Zouita et al., 2013	Athletes (*N*=16)	FIG=8 (21.56±2.27) NIG=8 (20.62±1.5)	8 weeks	3 days/20–30 min	NA	The Balance Master System Static Balance Assessment Isokinetic Measurement	Both groups showed an increase in maximal strength and a decrease in plantar flexion acceleration and deceleration times. However, better stability was observed in the injured group than in the healthy group.
Romero-Franco et al., 2013	Sprinter Athletes (*N*=33)	EG=16 (21.18±4.48) CG=17 (22.5 ±5.12)	6 weeks	3 days/30 min	NA	Squat Jump Test Countermovement Jump Test Stabiometry Test 30-meter Sprint Test	Proprioceptive training improved medial-lateral postural balance and jump performance in athletes. No improvement seen in speed.
Nikolaos et al., 2012	Basketball Players (*N*=26)	EG=13 (22.69±0.70) CG=13 (21.61±0.71)	12 weeks	NA	NA	Passing Assessment Test	Proprioception training improved passing technique skills in basketball players
Evangelos et al., 2012	Soccer Players (*N*=29)	EG= 15 (16.83±0.24) CG=14 (16.60±0.22)	10 Weeks	NA	NA	Jug 200, Jug Body 1, Jog Body 2 Tests Speed Dribbling Test Long and Short Passing Test Shooting Test	Proprioception training improved shot and long passing and jug ball technique skills in soccer players

EG: Exercise Group, PTG: Proprioceptive Training Group, MSTG= Muscular Strength Group, CST: Central Stabilization, EB: Experimental Boys, Experimental Girls: EG, IG: Intervention Group, Experimental Groups: EG, Control Groups: CG, Min: Minute, NA: Not Available, EGI: Eyes Group Exercise, FGI: Feet Group Exercise, FIG: Functional Instability Group, NIG: Non-Injured Group, SP: Soccer Players, BP: Basketball Players, TTG: Technical Training Group, BTG: Balance Training Group, PCG; Proprioceptive- Coordinative Group, RTG: Regular Training Group

### Physical and physiological performance responses

The results have shown that proprioceptive training enhances accurate passing skills, rapid shooting skills, and dynamic and static balance in basketball players [30], improves passing technique in athletes [31], enhances balance, explosive strength, agility, and dribbling skills in male soccer players [32], improves short passing, long passing, and ball bouncing skills in young male soccer players [33], enhances physiological capacity and technical skills in female soccer players [34], improves knee joint position sense and balance skills in male soccer and basketball players [35], and enhances passing and ball control techniques in male soccer players, but does not improve eye-hand coordination, muscle endurance, agility, sprint, and balance [36], improves static balance in male athletes [37], is effective in developing muscle activation and reducing chronic joint instability in handball players [38], improves balance in basketball players [39], enhances dynamic balance in soccer players [40], improves stance stability in taekwondo athletes [41], enhances postural stability in basketball players [42], improves dynamic balance in skaters [43], improve dynamic neuromuscular control in fencing [7], improves dynamic balance and service and passing technical skills in volleyball players [44], improves speed and agility in young soccer players but does not improve abdominal strength, balance, and flexibility [45], leads to an increase in maximal strength and a decrease in plantar flexion acceleration and deceleration times in athletes [46], and contributes to improvements in postural balance and jumping performance in track and field athletes [47].

## Discussion

This systematic review examined the effects of proprioceptive training on athletic performance. The importance of scientific findings that athletes use to prepare for sports competitions, such as national, international, and Olympic events, to achieve better performance is increasing. A literature review revealed that proprioceptive training improves athletes’ technical skills and physical performance in various disciplines. Zacharakis et al. [30] reported that proprioceptive training applied for eight weeks improved passing technique in both female and male basketball players and shooting technique in male players. A similar study found that a 12-week proprioceptive training program improved passing techniques in basketball players [31]. Different studies conducted on soccer players have shown that proprioceptive training improves dribbling skills in male soccer players [32], dribbling, passing, shooting, and heading skills in female soccer players [34], passing and ball control skills in young male soccer players [36], ball bouncing and long and short passing skills in young soccer players [33] and service and passing technical skills in youth female volleyball players [44]. Various studies have reported a positive correlation between proprioception and static and dynamic balance [48–50], balance and shooting technique proficiency [51], and balance, passing, and shooting technique proficiency [52]. Souglis et al. [34] noted that proprioceptive training programs have effectively improved coordination, balance, and proprioception, which are crucial for enhancing technical skills in sports. By improving proprioceptive acuity and dynamic neuromuscular control, athletes can enhance their motor skills and technical abilities, ultimately improving their respective sports performance [7]. Proprioceptive training positively affects athletes’ physiological attributes and technical skills, highlighting its importance for overall athletic performance [34]. Furthermore, proprioceptive training has been linked to improvements in muscle strength, joint stability, and postural balance, which are all essential for mastering technical skills in sports [53]. By improving proprioception and joint position sense, athletes can better control their movements, execute precise technical skills, and adapt to dynamic and challenging sports environments [54]. Proprioceptive training is a significant performance enhancement tool for athletes as it improves perception and coordination, which are essential for technical skills.

Various studies investigating the effects of proprioceptive training on athletes’ physical performance have shown improvements balance in female and male basketball players [30], male basketball players [39], professional soccer players [37, 40], young soccer players [32], youth female volleyball players [44], young speed skaters [43], fencers [7], male soccer and basketball players [35], female soccer players [55], taekwondo athletes [41], and sprinter track and field athletes [47]. Additionally, proprioceptive exercises have been found to enhance postural stability in young basketball players [42] and postural control in taekwondo athletes [56]. Proprioceptive training is essential to enhance athletes’ balance skills through several mechanisms. According to Han et al. [14], proprioceptive training programs effectively improve balance control by optimising ankle proprioceptive information reweighting, which is beneficial for sports performance and injury prevention. These programs have been found to reduce the incidence of ankle sprains in athletes, highlighting the importance of proprioceptive training for maintaining joint stability and preventing injuries [12]. Additionally, proprioceptive training can improve dynamic neuromuscular control in athletes, further emphasising its positive effect on balance skills [7]. Athletes with enhanced proprioceptive acuity and muscle strength, often developed through long-term athletic training, exhibit superior balance abilities compared to non-athletes. This enhanced proprioception contributes to better balance control and coordination, essential for optimal athletic performance. Moreover, proprioceptive training has been linked to improvements in static and dynamic balance values in elite and amateur soccer players, indicating its positive effects on balance ability in athletes [36]. Additionally, studies have shown that neuromuscular training regimens, including protocols that challenge strength, agility, and balance, can significantly improve balance and proprioception in athletes [57]. Moreover, balance training in proprioceptive training has been emphasised as crucial for enhancing performance attributes such as agility and balance, which are essential for executing technical movements precisely and efficiently [58]. Generally, proprioceptive exercises consist of standing on a balance board or ankle disk, standing in balance with eyes closed, throwing and catching a ball while standing on one leg, and dribbling a ball [59]. These exercises can help improve balance skills by activating and strengthening lower extremity muscles.

Several studies have explored the effects of proprioceptive training on athletes and have found that such training enhances speed and agility in male soccer players [45], agility in male soccer players [32, 60], and agility in female soccer players [55]. Additionally, it improves jumping ability in sprinter track and field athletes [47] and increases maximal strength in athletes [46]. Following proprioceptive training, enhanced balance contributes to better stability during complex movements such as acceleration, deceleration, and change of direction, thus making acceleration and quick directional changes easier [32]. Gruber and Golhoffer [61] pointed out that the swift transition from eccentric to concentric muscle contractions in vertical jumps influences explosive force development and that proprioceptive training may involve a higher rate of motor units, potentially affecting rapid power generation. Winter et al. [62] showed that proprioceptive training can improve both proprioceptive and motor performance, with similar advancements in both areas. This indicates that proprioceptive training can comprehensively affect motor skills, encompassing both the sensory and motor aspects of movement. Consequently, it can be asserted that proprioceptive training positively affects speed, agility, and jumping performance, primarily owing to its effect on rapid power generation, acceleration, and smoother changes in direction.

Other studies have shown that proprioceptive training is efficacious in improving muscle activation, reducing chronic joint instability in handball players [38], reducing plantar flexion acceleration and deceleration times in track and field athletes [46], enhancing knee joint position sense in male soccer and basketball players [35], and increasing the physiological capacity of athletes [34]. Enhanced proprioceptive abilities contribute to good joint stability, thus ensuring positive stimulus-response synchronisation, which helps prevent injuries [63]. Park et al. [64] indicated that developing balance and proprioceptive function through exercise can maximize exercise capacity and promote development in joint stability for both athletes and sedentary individuals. A previous study demonstrated that proprioceptive training could lead to rapid learning gains in proprioceptive acuity and untrained motor performance at the practised joint [4]. Proprioceptive training is essential to enhance motor skills in athletes through various mechanisms. Highly trained athletes exhibit enhanced proprioceptive acuity and muscle strength, which can be inherent or developed through long-term athletic training [65]. This improved proprioceptive function enhances motor function, coordination, and, ultimately, better athletic performance [66]. Proprioceptive training programs have stabilised joints, prevented injuries, and enhanced postural balance in athletes [7]. By improving proprioception, athletes can control their movements, maintain balance, and execute precise motor skills required in various sports disciplines [67]. Furthermore, proprioceptive exercises can enhance stability, particularly in lower extremity joints, and protect athletes against injuries.

In recent years, with an increased competitive environment for superior performance, athletes must outperform their opponents in strength, speed, endurance, and stress coping, which can increase the risk of injury [68]. In addition, well-developed proprioceptive abilities provide positive stimulus-response synchronisation and achieve good performance in joint stability to prevent injuries [63]. The relationship between excellent sports performance and proprioceptive ability suggests that coaches should include proprioceptive training in their programs [69].

Proprioception and the vestibular sense are distinct but complementary systems that contribute to balance and coordination. Proprioception, the body’s ability to perceive its own position and movement, is mediated by sensory receptors in muscles, tendons, and joints [70]. Vestibular sense, on the other hand, involves the inner ear’s semicircular canals and otolith organs, which detect head movements and help maintain balance and spatial orientation [71]. Both systems are crucial for balance and coordination, with proprioceptive training focusing on enhancing the sensory feedback loop from the musculoskeletal system, and vestibular training targeting the inner ear’s balance mechanisms [72]. The integration of these systems is essential for the brain’s predictive sensing of head movement during voluntary self-motion [71].

## Conclusion

This systematic review assessed the effects of proprioceptive training on athletic performance by analyzing 19 studies involving 621 participants from various sports disciplines. The findings demonstrate that proprioceptive training significantly enhances multiple aspects of athletic performance, although the extent of these effects may vary based on factors such as sex, age, and type of sport.

Key findings include:

1. **Balance and Postural Stability**: Nine studies indicated that proprioceptive training significantly enhances balance and postural stability, crucial for reducing injury risk and improving overall athletic performance.
2. **Physiological Capacities and Technical Skills**: Three studies reported improvements in physiological capacities, such as VO2 Max and body fat ratio, as well as in technical skills like dribbling, passing, and shooting.
3. **Explosive Power and Muscle Activation**: Four studies found positive effects on explosive power, muscle activation, and general strength, essential for sports requiring sudden bursts of strength and speed.
4. **Agility and Speed**: Three studies showed that proprioceptive training improves agility and speed, enhancing an athlete’s ability to make rapid movements and direction changes.
5. **Muscle Coactivation and Joint Stability**: One study highlighted a reduction in muscle coactivation and chronic joint instability, further supporting the role of proprioceptive training in injury prevention.

In conclusion, proprioceptive training is a valuable component of athletic training programs, as it enhances proprioceptive acuity, muscle strength, motor function, coordination, and balance. By incorporating proprioceptive exercises into their routines, athletes can optimize their motor skills, reduce injury risks, and improve overall performance.

While the existing literature underscores the importance of proprioceptive training, there remains a need for further research to identify optimal training strategies, applications, and outcomes. Future studies should explore the effectiveness of proprioceptive training across different sports, refine training protocols, and examine its impact on specific injury patterns and performance metrics. Integrating these insights can help coaches and athletes develop more effective training regimens that maximize performance and minimize injury risks.

### Electronic supplementary material

Below is the link to the electronic supplementary material.

Supplementary Material 1

Supplementary Material 2

## Data Availability

The dataset supporting the conclusions of this article is included within the article.
