# Supplementary material for: Effects of proprioceptive training on sports performance: a systematic review
Source: BMC Sports Sci Med Rehabil. 2024 Jul 4;16:149. doi: 10.1186/s13102-024-00936-z (PMC11225257; doi:10.1186/s13102-024-00936-z)
Supplement: Supplementary file 1 — Supplementary Material 1 [file 13102_2024_936_MOESM1_ESM.pdf]

Systematic review

*Fields that have an **asterisk (*)** next to them means that they **must be answered. Word limits** are provided for each section. You will be unable to submit the form if the word limits are exceeded for any section. Registrant means the person filling out the form.*

1. * Review title.

Give the title of the review in English

Effects of Proprioceptive Training on Sports Performance: A Systematic Review

2. Original language title.

For reviews in languages other than English, give the title in the original language. This will be displayed with the English language title.

English

3. * Anticipated or actual start date.

Give the date the systematic review started or is expected to start.

01/01/2010

4. * Anticipated completion date.

Give the date by which the review is expected to be completed.

01/10/2022

5. * Stage of review at time of this submission.

This field uses answers to initial screening questions. It cannot be edited until after registration.

Tick the boxes to show which review tasks have been started and which have been completed.

Update this field each time any amendments are made to a published record.

The review has not yet started: Yes

Review stage	Started	Completed
Preliminary searches	No	No
Piloting of the study selection process	No	No
Formal screening of search results against eligibility criteria	No	No
Data extraction	No	No
Risk of bias (quality) assessment	No	No
Data analysis	No	No

Provide any other relevant information about the stage of the review here.

6. * Named contact.

The named contact is the guarantor for the accuracy of the information in the register record. This may be any member of the review team.

Osman Yılmaz

Email salutation (e.g. "Dr Smith" or "Joanne") for correspondence:
Dr Yılmaz

7. * Named contact email.

Give the electronic email address of the named contact.

osmanyilmaz@osmaniye.edu.tr

8. Named contact address

PLEASE NOTE this information will be published in the PROSPERO record so please do not enter private information, i.e. personal home address

Give the full institutional/organisational postal address for the named contact.

Osmaniye Korkut Ata University, 80000 Osmaniye, Turkey

9. Named contact phone number.

Give the telephone number for the named contact, including international dialling code.

+905329529227

10. * Organisational affiliation of the review.

Full title of the organisational affiliations for this review and website address if available. This field may be completed as 'None' if the review is not affiliated to any organisation.

Osmaniye Korkut Ata University

Organisation web address:

11. * Review team members and their organisational affiliations.

Give the personal details and the organisational affiliations of each member of the review team. Affiliation refers to groups or organisations to which review team members belong.
NOTE: email and country now MUST be entered for each person, unless you are amending a published record.

Dr Osman Yılmaz. Osmaniye Korkut Ata University, School of Physical Education and Sports, Osmaniye, Turkey

Assistant/Associate Professor Yusuf Soylu. Tokat Gaziosmanpasa University, Faculty of Sports Sciences, Tokat, Turkey

Professor Nurtekin Erkmen. Selcuk University, Faculty of Sports Sciences, Konya, Türkiye

Professor Turgut Kaplan. None Faculty

Dr Ladislav Batalik. Department of Physiotherapy and Rehabilitation, Faculty of Medicine, Masaryk University, Brno, Czech Republic

12. * Funding sources/sponsors.

Details of the individuals, organizations, groups, companies or other legal entities who have funded or sponsored the review.

None

Grant number(s)

State the funder, grant or award number and the date of award

None

13. * Conflicts of interest.

List actual or perceived conflicts of interest (financial or academic).

None

14. Collaborators.

Give the name and affiliation of any individuals or organisations who are working on the review but who are not listed as review team members. **NOTE: email and country must be completed for each person, unless you are amending a published record.**

15. * Review question.

State the review question(s) clearly and precisely. It may be appropriate to break very broad questions down into a series of related more specific questions. Questions may be framed or refined using PI(E)COS or similar where relevant.

Does proprioceptive training affect sports performance?

16. * Searches.

State the sources that will be searched (e.g. Medline). Give the search dates, and any restrictions (e.g. language or publication date). Do NOT enter the full search strategy (it may be provided as a link or attachment below.)

Web of Science, PubMed, and Scopus

17. URL to search strategy.

Upload a file with your search strategy, or an example of a search strategy for a specific database, (including the keywords) in pdf or word format. In doing so you are consenting to the file being made publicly accessible.

Or provide a URL or link to the strategy. Do NOT provide links to your search **results**.

https://www.crd.york.ac.uk/PROSPEROFILES/530268_STRATEGY_20240329.pdf

Do not make this file publicly available until the review is complete

18. * Condition or domain being studied.

Give a short description of the disease, condition or healthcare domain being studied in your systematic review.

This study presents a systematic review of peer-reviewed scientific articles investigating the effects of proprioceptive training on athletic performance characteristics in athletes.

19. * Participants/population.

Specify the participants or populations being studied in the review. The preferred format includes details of both inclusion and exclusion criteria.

Keywords were grouped into two distinct categories: those designed to retrieve studies utilizing proprioception intervention as an exercise technique or objective (encompassing terms like "central stabilization," "eyes group exercise," and "feet group exercise") and those focused on the balance or injury risk of proprioception intervention. Second, the proprioception training's physiological, physical, and technical ability outcomes include dribbling, explosive power, and strength. This study used "proprioceptive training" and "proprioceptive exercise."

20. * Intervention(s), exposure(s).

Give full and clear descriptions or definitions of the interventions or the exposures to be reviewed. The preferred format includes details of both inclusion and exclusion criteria.

Proprioception enhances sports performance by allowing athletes better control and awareness of their body movements and positions. The role of proprioception in improving sports performance is to provide athletes with a heightened sense of body awareness and control. It allows athletes to make precise and coordinated movements, adjust their body positions in response to changing demands, and maintain optimal balance and stability. The current systematic review was conducted using standardized search operators that included the presence of proprioceptive intervention on sports performance or proprioceptive intervention on athletic performance.

21. * Comparator(s)/control.

Where relevant, give details of the alternatives against which the intervention/exposure will be compared (e.g. another intervention or a non-exposed control group). The preferred format includes details of both inclusion and exclusion criteria.

In the articles included in the research, Proprioceptive Training training application was compared with Muscular Strength Group Central Stabilization, Functional Instability Group, Technical Training Group and Regular Training Group.

22. * Types of study to be included.

Give details of the study designs (e.g. RCT) that are eligible for inclusion in the review. The preferred format includes both inclusion and exclusion criteria. If there are no restrictions on the types of study, this should be stated.

This study presents a systematic review of peer-reviewed scientific articles investigating the effects of proprioceptive training on athletic performance characteristics in athletes. The review focused solely on studies examining the impact of proprioceptive interventions or training on athletes' sports performance, encompassing functional and structural aspects within their research protocols. Only experimental studies were included in this study. No limits were placed on the sex, age, sports type, or athletic level of eligible articles. This study included only athletic participants. We had articles that utilized applied methodologies to provide a perspective.

23. Context.

Give summary details of the setting or other relevant characteristics, which help define the inclusion or exclusion criteria.

This systematic review aims to evaluate the effectiveness of proprioceptive training intervention type in athletes.

24. * Main outcome(s).

Give the pre-specified main (most important) outcomes of the review, including details of how the outcome is defined and measured and when these measurement are made, if these are part of the review inclusion criteria.

Following the search, 170 articles were identified using relevant keywords, of which 16 directly addressed sports performance and were included in this study. The findings revealed that proprioceptive training had a positive influence on various aspects of athletic performance, including physiological capacity, balance, explosive strength, speed, agility, postural stability, knee joint position sense, muscle activation, reduction of chronic joint instability, dribbling, passing, and technical ball-control skills.

Measures of effect

25. * Additional outcome(s).

List the pre-specified additional outcomes of the review, with a similar level of detail to that required for main outcomes. Where there are no additional outcomes please state ‘None’ or ‘Not applicable’ as appropriate to the review

None

Measures of effect

26. * Data extraction (selection and coding).

Describe how studies will be selected for inclusion. State what data will be extracted or obtained. State how this will be done and recorded.

A systematic review of the literature was performed following the Preferred Reporting Items for Systematic Reviews and Meta-Analyses (PRISMA) guidelines. After obtaining the article results from the databases, the titles and abstracts were examined to identify articles related to the keywords. The criteria for including articles in this review were determined using the PICO (Population, Intervention, Comparison, Outcome) approach.

27. * Risk of bias (quality) assessment.

State which characteristics of the studies will be assessed and/or any formal risk of bias/quality assessment tools that will be used.

The quality assessment of the included articles was performed using the Testex scale criteria. Scores below four were considered "poor quality," scores between 4 and 7 were considered "moderate quality," scores between 8 and 11 were considered "good quality," and scores above 11 were considered "excellent quality".

28. * Strategy for data synthesis.

Describe the methods you plan to use to synthesise data. This **must not be generic text** but should be **specific to your review** and describe how the proposed approach will be applied to your data.
If meta-analysis is planned, describe the models to be used, methods to explore statistical heterogeneity, and software package to be used.

None

29. * Analysis of subgroups or subsets.

State any planned investigation of 'subgroups'. Be clear and specific about which type of study or participant will be included in each group or covariate investigated. State the planned analytic approach.

None

30. * Type and method of review.

Select the type of review, review method and health area from the lists below.

Type of review

Cost effectiveness	No
Diagnostic	No
Epidemiologic	No
Individual patient data (IPD) meta-analysis	No
Intervention	No
Living systematic review	No
Meta-analysis	No
Methodology	No
Narrative synthesis	No
Network meta-analysis	No
Pre-clinical	No
Prevention	No
Prognostic	No
Prospective meta-analysis (PMA)	No
Review of reviews	No
Service delivery	No

Synthesis of qualitative studies	No
Systematic review	Yes
Other	No

Health area of the review

Alcohol/substance misuse/abuse	No
Blood and immune system	No
Cancer	No
Cardiovascular	No
Care of the elderly	No
Child health	No
Complementary therapies	No
COVID-19	No
Crime and justice	No
Dental	No
Digestive system	No
Ear, nose and throat	No
Education	No
Endocrine and metabolic disorders	No
Eye disorders	No
General interest	No
Genetics	No
Health inequalities/health equity	No
Infections and infestations	No
International development	No
Mental health and behavioural conditions	No
Musculoskeletal	Yes
Neurological	No
Nursing	No
Obstetrics and gynaecology	No

Oral health	No
Palliative care	No
Perioperative care	No
Physiotherapy	Yes
Pregnancy and childbirth	No
Public health (including social determinants of health)	No
Rehabilitation	Yes
Respiratory disorders	No
Service delivery	No
Skin disorders	No
Social care	No
Surgery	No
Tropical Medicine	No
Urological	No
Wounds, injuries and accidents	No
Violence and abuse	No

31. Language.

Select each language individually to add it to the list below, use the bin icon to remove any added in error.

English

There is an English language summary.

32. * Country.

Select the country in which the review is being carried out. For multi-national collaborations select all the countries involved.

Türkiye

33. Other registration details.

Name any other organisation where the systematic review title or protocol is registered (e.g. Campbell, or The Joanna Briggs Institute) together with any unique identification number assigned by them.
If extracted data will be stored and made available through a repository such as the Systematic Review Data Repository (SRDR), details and a link should be included here. If none, leave blank.

Ethical approval is not required for review articles

34. Reference and/or URL for published protocol.

If the protocol for this review is published provide details (authors, title and journal details, preferably in Vancouver format)

Moher D, Shamseer L, Clarke M, Ghersi D, Liberati A, Petticrew M. Prisma-P Group. Preferred reporting items for systematic review and meta-analysis protocols (PRISMA-P) 2015 statement. Syst. Rev. 2015;4, 1-9.
https://doi:10.1186/2046-4053-4-1

Huang X, Lin J, Demner-Fushman D. Evaluation of PICO as a knowledge representation for clinical questions. American Medical Informatics Association. p. 2006;359

https://link.springer.com/article/10.1186/2046-4053-4-1
https://PubMed.ncbi.nlm.nih.gov/17238363/

No I do not make this file publicly available until the review is complete

35. Dissemination plans.

Do you intend to publish the review on completion?

Yes

We plan to disseminate the findings of this systematic review through various channels. We aim to publish the review, preferably in an open access scientific journal.

36. Keywords.

Give words or phrases that best describe the review. Separate keywords with a semicolon or new line. Keywords help PROSPERO users find your review (keywords do not appear in the public record but are included in searches). Be as specific and precise as possible. Avoid acronyms and abbreviations unless these are in wide use.

Athlete, Athletic performance, Proprioceptive training, Proprioceptive exercise, proprioception

37. Details of any existing review of the same topic by the same authors.

If you are registering an update of an existing review give details of the earlier versions and include a full bibliographic reference, if available.

No

38. * Current review status.

Update review status when the review is completed and when it is published.
New registrations must be ongoing so this field is not editable for initial submission.

Review_Ongoing

39. Any additional information.

Provide any other information relevant to the registration of this review.

None

40. Details of final report/publication(s) or preprints if available.

Leave empty until publication details are available OR you have a link to a preprint (NOTE: this field is not editable for initial submission).
List authors, title and journal details preferably in Vancouver format.
